# Acvr2b receptors transduce all BMP signaling in the zebrafish gastrula and restrict Fibrodysplasia Ossificans Progressiva ACVR1-R206H signaling in a dose-dependent manner

**DOI:** 10.1101/2025.10.07.680911

**Published:** 2025-10-07

**Authors:** Jeet H. Patel, Benjamin Tajer, Jira White, Finn Warrick, Mary C. Mullins

**Affiliations:** 1 Department of Cell and Developmental Biology, University of Pennsylvania Perelman School of Medicine, Philadelphia, PA, United States

## Abstract

BMP signaling drives dorsoventral (DV) axial patterning in vertebrates and invertebrates, with BMP dimers recruiting tetrameric receptor complexes to phosphorylate SMADs that activate ventral target gene expression. In zebrafish DV patterning, BMP2/7 heterodimers exclusively signal, assembling a receptor complex of two distinct type I receptors, Bmpr1 and Acvr1l, that canonically bind Bmp2 and Bmp7 ligands, respectively. We tested if the two distinct classes of BMP type II receptors, Bmpr2 and Acvr2, also act in the signaling complex. We determined that Acvr2 receptors solely transduce BMP signaling in DV patterning. We mutated all four *acvr2a* and *acvr2b* genes in the zebrafish and found that maternal-zygotic depletion of just Acvr2b receptors abrogates all BMP signaling, indicating that Acvr2b is the primary type II receptor transducing BMP signaling in the gastrula. We further demonstrated that hyperactive signaling through the ACVR1-R206H Fibrodysplasia Ossificans Progressiva human disease-causing mutant receptor is restricted when maternal and zygotic contributions of either Acvr2ba or Acvr2bb are absent. This reveals an increased sensitivity of ACVR1-R206H signaling to Acvr2b dosage, compared to wild-type ACVR1. These findings support a model in which Acvr2b receptors mediate the endogenous BMP signaling in the gastrula and that hyperactivity of ACVR1-R206H is limited in a dose-dependent manner by the relative concentration of Acvr2b.

## INTRODUCTION

BMP signaling functions as a major pathway in embryonic development, regulating patterning of the dorsoventral (DV) axis, neural tube, and digits in addition to vascular development and angiogenesis (Chen et al., 2004; Wang et al., 2014; Yan and Wang, 2021; Zinski et al., 2018). The general mechanism of BMP signaling is well defined. BMP ligands are secreted as dimers, binding a serine-threonine kinase receptor tetramer consisting of two type I and two type II receptors. Upon ligand binding, the constitutively active type II receptor kinase phosphorylates the serine/threonine rich domain (GS domain) of the type I receptor, activating it. Active type I receptors phosphorylate intracellular SMAD transcription factors, which then accumulate in the nucleus and regulate target gene expression (Chen et al., 2004; Mueller and Nickel, 2012; Yadin et al., 2016).

In zebrafish, the BMP signaling components required for DV axial patterning are well defined. In the gastrula, Bmp2/7 heterodimers signal through a heteromeric receptor complex consisting of the type I receptors Acvr1l and Bmpr1a, and two type II receptors (Bauer et al., 2001; Little and Mullins, 2009; Mintzer et al., 2001; Mullins et al., 1996; Tajer et al., 2021). Interestingly, these two type I receptors have been sub-functionalized in signaling in the gastrula, with Acvr1l kinase activity essential for signaling, while Bmpr1a kinase activity is dispensable (Tajer et al., 2021). To further determine how heterodimers and heteromeric type I receptor complexes signal, it is critical to understand the type II receptors regulating signaling.

The BMP type II receptors acting in zebrafish axial patterning are not fully defined. The two distinct classes of type II receptors, Acvr2 and Bmpr2, are represented by six genes in the zebrafish; Acvr2 receptors (*acvr2aa*, *acvr2ab*, *acvr2ba*, *acvr2bb*) that mediate signaling by both BMP and Nodal, and the BMP specific Bmpr2 receptors (*bmpr2a* and *bmpr2b*). Morpholino (MO) or *acvr2* quadruple F0 CRISPR knockdowns result in moderate BMP loss-of-function phenotypes (Albertson et al., 2005; Preiß et al., 2022), indicating that Acvr2 receptors function in BMP signaling. Neither F0 CRISPants nor stable zygotic mutants completely abrogated BMP signaling, indicating that additional type II receptors act in transducing BMP signaling during DV patterning. One possibility is that Bmpr2 receptors function partially redundantly to Acvr2; however, whether *bmpr2* is expressed is unclear due to conflicting reports (Monteiro et al., 2008; White et al., 2017). Both MO knockdown and double zygotic *bmpr2a^−/−^*;*bmpr2b^−/−^* mutants do not display axial patterning defects (Monteiro et al., 2008; Zhang et al., 2020). As such, maternally-supplied Bmpr2 or Acvr2 receptor pools could be sufficient for early embryonic DV patterning, as is the case for Acvr1l, Smad5, and Bmp1a (Kramer et al., 2002; Mintzer et al., 2001; Tuazon et al., 2020). Determining the type II receptor sources is essential to investigating mechanisms by which heteromeric receptor complexes signal in the gastrula.

In the mouse, the three type II receptors, ACVR2A, ACVR2B, and BMPR2, are required in early development. *Bmpr2* mouse mutants are embryonic lethal by E9.5, arresting prior to gastrulation (Beppu et al., 2000), similar to *Bmp4*, *Acvr1*, and *Bmpr1* mutants, indicating a requirement for BMPR2 in early BMP signaling (Gu et al., 1999; Mishina et al., 1995; Winnier et al., 1995). Single mutants of *Acvr2a* or *Acvr2b* do not exhibit embryonic phenotypes (Matzuk et al., 1995; Oh and Li, 1997). However, *Acvr2a^−/−^*;*Acvr2b^−/−^* double mutants arrest development by E8.5, indicating that they function redundantly in signaling in early mouse development (Song et al., 1999). As *Bmpr2* and double *Acvr2a^−/−^*;*Acvr2b^−/−^* mutants display early embryonic lethality, these two classes of type II receptor are likely not redundant with each other at these stages.

Defining specific requirements for type II receptors in signaling has key implications for modeling of the autosomal dominant disease known as fibrodysplasia ossificans progressiva (FOP). Over 90% of FOP patients have a recurrent mutation in the GS domain of ACVR1, R206H (Hüning and Gillessen-Kaesbach, 2014; Shore et al., 2006) that hyperactivates BMP signaling due to increased Activin and BMP responsiveness (Hatsell et al., 2015; Hildebrand et al., 2017). ACVR1-R206H expression in zebrafish embryos causes embryonic ventralization and increased BMP signaling via Smad5 (Allen et al., 2020; Mucha et al., 2018; Shen et al., 2009). Interestingly, FOP mutant receptors display ligand-independent activity, though still require type II receptors for signaling (Allen et al., 2020; Hildebrand et al., 2017; Le and Wharton, 2012; Shen et al., 2009). In the mouse either ACVR2A or BMPR2 must be present for signaling (Bagarova et al., 2013). In zebrafish, MO knockdown of Acvr2ba and Acvr2bb, but not of Acvr2a or Bmpr2, reduced R206H mediated signaling, though did not block it (Lalonde et al., 2023).

Here, we interrogate the type II receptor requirements for axial patterning and FOP signaling in the zebrafish. We find that *bmpr2* is not expressed strongly, if at all, prior to gastrulation, leaving Acvr2a and Acvr2b as possible type II receptors. By examining mutant alleles for *acvr2aa*, *acvr2ab*, *acvr2ba*, and *acvr2bb*, we identify both maternal and zygotic contributions of each gene to embryonic patterning. We show that zygotic loss of *acvr2* genes leads to minor dorsalization, though not a complete loss of BMP signaling. We find maternal and zygotic depletion of both *acvr2ba* and *acvr2bb* abrogates all BMP signaling, leading to complete embryonic dorsalization, and demonstrating that Acvr2b receptors transduce all BMP signaling in the gastrula. Finally, we find the maternal and zygotic contribution of Acvr2ba and Acvr2bb is needed for signaling via human ACVR1-R206H, showing that signaling by ACVR1-R206H requires a higher Acvr2b dose than endogenous BMP signaling in DV axial patterning, where either Acvr2ba or Acvr2bb is sufficient for signaling.

## RESULTS

### Bmpr2 is not expressed in the early embryo

**Figure 1:**
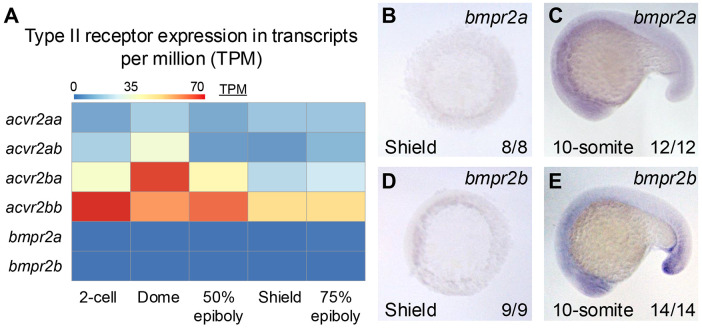
Bmpr2 receptors are not strongly expressed in the early embryo A) Expression of type II receptor genes in transcripts per million (TPM) for all six type II receptors genes ranging from 2-cell (exclusively maternal) to 75% epiboly, when axial patterning is underway (White et. al, 2017). B-E) *in situ* hybridization at shield (B,D) and 10-somite (C,E) for *bmpr2a* (B,C) *bmpr2b* (D,E).Number of embryos displaying the observed phenotype of the total assayed, representative of 2 experiments.

We first set out to determine which type II BMP receptors were present in the early embryo. All four *acvr2* genes are expressed at the 2-cell stage (maternal deposition) and through shield stage, based on both *in situ* hybridization (Preß et al., 2022) and RNA-seq data (White et al., 2017) (Figure 1A). Previous *in situ* hybridization analysis found that *bmpr2a* and *bmpr2b* were expressed in embryos ranging from the one-cell stage through 52 hours post fertilization (Monteiro et al., 2008). However, neither *bmpr2* transcript was detected in early stage transcriptomic data sets (White et al., 2017) (Figure 1A). To address this discrepancy, we performed *in situ* hybridization for *bmpr2a* and *bmpr2b* at both early gastrula and 17-somite stages (Figure 1B-E). At 17-somites, both *bmpr2a* and *bmpr2b* are expressed in the head and tail, with *bmpr2b* also detected in the proctodeum (Figure 1C,E), in agreement with prior reports (Monteiro et al., 2008). However, we did not detect *bmpr2a* or *bmpr2b* at early gastrulation, in agreement with the RNA-seq data (Figure 1A-C). We conclude that Bmpr2 is not expressed in the early embryo and therefore cannot participate in axial patterning.

### Maternal loss of Acvr2 increases severity of zygotic loss-of-function phenotypes

**Figure 2:**
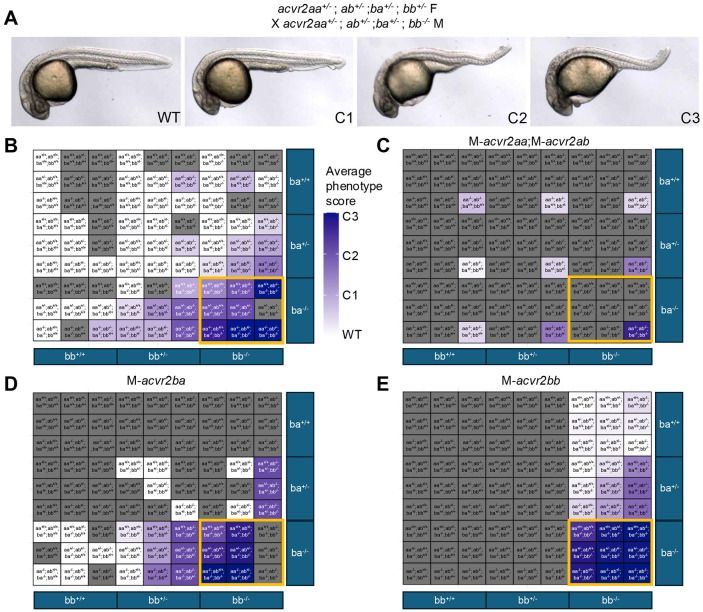
Maternal-Zygotic loss of Acvr2 causes embryonic dorsalization A) Phenotypes observed in Acvr2 mutants based on established dorsalization criteria at 1dpf. Compared to WT, C1 have a reduced ventral tail fin, C2 have complete loss of ventral tail fin and ventral tail vein, and C3 display C2 phenotypes and fail to extend their yolk extension. B-E) Average dorsalization scores for each genotype based on assayed embryos. White represents 100% WT (scored 0), and dark blue represents 100% C3 (score 3), with C1 and C2 scored as 1 or 2, respectively. Grey indicates no embryos of such genotype(s). Maternal genotypes were: no homozygous maternal mutations (B), M-*acvr2aa^−/−^*;M-*acvr2ab^−/−^* (C), M-*acvr2ba^−/−^* (D) M-*acvr2bb^−/−^* (E). Orange boxes highlight *acvr2ba^−/−^;acvr2bb^−/−^* double mutants. Parental genotypes for crosses are as follows (female X male genotype): B) *acvr2aa^+/−^*;*ab^+/+^;ba^+/−^*;*bb^+/−^* X *acvr2aa^+/−^*;*ab^+/+^;ba^+/−^*;*bb^+/−^* ; *acvr2aa^+/−^*;*ab^+/−^;ba^+/−^*;*bb^+/−^* X *acvr2aa^+/−^*;*ab^+/−^;ba^+/−^*;*bb^+/−^* ; *acvr2aa^+/−^*;*ab^+/−^;ba^+/−^*;*bb^+/−^* X *acvr2aa^+/−^*;*ab^+/−^;ba^+/−^*;*bb^−/−^* OR *acvr2aa^+/−^*;*ab^+/−^;ba^+/−^*;*bb^+/−^* X *acvr2aa^−/−^*;*ab^−/−^;ba^+/−^*;*bb^+/−^* ; C) *acvr2aa^−/−^*;*ab^−/−^;ba^+/−^*;*bb^+/−^* X *acvr2aa^−/−^*;*ab^−/−^;ba^+/−^*;*bb^+/−^* ; D) *acvr2aa^+/−^*;*ab^+/−^;ba^−/−^*;*bb^+/−^* X *acvr2aa^+/−^*;*ab^+/−^;ba^−/−^*;*bb^+/−^* OR *acvr2aa^+/−^*;*ab^−/−^;ba^+/−^*;*bb^−/−^* ; *acvr2aa+/−*;*ab^+/+^;ba−/−*;*bb^+/−^* X *acvr2aa^+/−^*;*ab^+/−^;ba^+/−^*;*bb^−/−^* ; *acvr2aa^+/−^*;*ab^+/−^;ba^−/−^*;*bb^+/+^* X *acvr2aa^+/−^*;*ab^+/−^;ba^−/−^*;*bb^+/+^* ; E) *acvr2aa^+/−^*;*ab^+/−^;ba^+/−^*;*bb^−/−^* X *acvr2aa^+/−^*;*ab^+/−^;ba^+/−^*;*bb^−/−^* ;

We next asked if maternal *acvr2* expression contributes to BMP signaling in gastrula DV patterning, specifically if maternal receptor expression could explain partial BMP signaling seen in zygotic *acvr2* CRISPants/mutants (Preiß et al., 2022). We obtained the sanger allele *acvr2ab^sa18285^* and generated mutant alleles of *acvr2aa*, *acvr2ba*, and *acvr2bb*. All 4 alleles result in frameshifts, terminating in premature stop codons (Supplemental Figure S1). We generated combinations of these alleles to test the maternal and zygotic contributions of each gene to embryonic patterning. Phenotypes were assessed blind to genotypes at 1 day post fertilization (dpf) based on previously established dorsalized staging criteria (Figure 2A) (Mullins et al., 1996), then each individual was genotyped. We genotyped all mutant embryos and, as most embryos from these crosses were phenotypically wild type, we did not always genotype each wild-type embryo.

We found that multiple *acvr2* receptors must be mutant to cause embryonic dorsalization (Figure 2B). Single and double mutants for *acvr2aa*, *acvr2ab*, and *acvr2ba* were mostly wild type, with weak dorsalization when coupled with *acvr2bb*^+/−^ (Figure 2B, middle 3 columns). Triple *acvr2aa^−/−^*;*acvr2ab^−/−^*;a*cvr2ba^−/−^* mutants display weak dorsalization (C1-C2), with moderate C3 phenotypes seen in some quadruple mutants (Figure 2B, lower right box). *acvr2bb^−/−^* single mutants were wild type but resulted in minor dorsalization with increasing numbers of other mutated *acvr2* genes (Figure 2B, right 3 columns). Notably, the strongest phenotypes were generally observed in *acvr2ba^−/−^*;a*cvr2bb^−/−^*, suggesting that Acvr2b receptors contribute most strongly to signaling (Figure 2B, orange border).

Maternal loss of Acvr2 receptors enhanced patterning defects. Loss of maternal *acvr2aa* and *acvr2ab* (M-*acvr2aa^−/−^*;M-*acvr2ab^−/−^*) resulted in slightly more severe dorsalization for most genotypes, though the strongest phenotypes still required zygotic loss of both Acvr2b receptors (Figure 2C, orange border). As zygotic mutants for Acvr2b receptors were more strongly dorsalized than those for Acvr2a, we focused on maternal loss of either Acvr2ba or Acvr2bb. M-*acvr2ba^−/−^* or M-*acvr2bb^−/−^* resulted in stronger dorsalization for most genotypes (Figure 2D,E). Specifically, *acvr2ba^−/−^*;*acvr2bb^−/−^* double mutant embryos with maternal loss of either Acvr2b gene displayed stronger, C3 dorsalized phenotypes regardless of other mutations (Figure 2D,E, orange border). These findings indicate that maternal loss of Acvr2 receptors increases the severity of phenotypes, suggesting a significant contribution of maternal Acvr2ba and Acvr2bb for early BMP signaling in the embryo.

### Acvr2ba and Acvr2bb are the predominant type II receptors for BMP signaling during DV patterning

**Figure 3:**
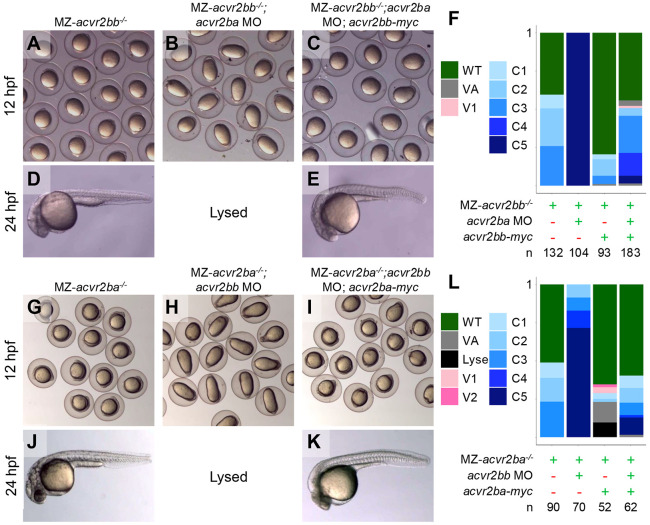
Maternally contributed Acvr2ba and Acvr2bb are redundant in DV patterning Representative images of maternal-zygotic (MZ) *acvr2bb* and *acvr2ba* mutants at 12 and 24 hpf (A,D,G,J). Following injection with morpholino for the reciprocal gene (2.5-5ng *acvr2ba* MO or *acvr2bb* MO mix (2.5ng *acvr2bb* UTR MO with 5 ng *acvr2bb* ATG MO), embryos are elongated by 12hpf and lyse, indicative of strong C5 dorsalized phenotypes (B,H). MZ-*acvr2bb* can be rescued with 10-30 pg of *acvr2bb* mRNA (C,E) and MZ-*acvr2ba* can be rescued with 50 pg *acvr2ba* mRNA (I,K). Head necrosis can be seen in MZ-*acvr2ba* embryos injected with *acvr2bb* MO and rescued (K), likely due to toxicity of the *acvr2bb* MO. Proportions of embryos with different phenotypes in each condition (F,L). Data in F is from 6 independent experiments (expect for *acvr2bb-myc* injection into MZ-*acvr2bb* mutants, which is from 3 independent experiments). Data in L is from 3 independent experiments.

Maternal loss of a single Acvr2b gene did not fully dorsalize embryos, as predicted if Acvr2b is the predominant type II receptor required for signaling. Therefore, we asked if combined maternal and zygotic loss of both Acvr2b receptors could completely abrogate BMP signaling. Zygotic *acvr2ba^−/−^*;*acvr2bb^−/−^* fish are not viable to adulthood, preventing generation of double maternal-zygotic (MZ) *acvr2ba^−/−^*;MZ-*acvr2bb^−/−^* embryos. Instead, we crossed maternal mutants of either gene and injected morpholinos (MO) targeting the reciprocal gene to prevent translation of maternally deposited transcripts. Knockdown of Acvr2ba in MZ-*acvr2bb^−/−^* embryos resulted in the strongest C5 dorsalization phenotype, indicative of complete loss of BMP signaling (Figure 3A,B,D,F). For the reciprocal experiment, we injected MZ-*acvr2bb^−/−^* embryos with a mix of published ATG *acvr2bb* MO (Dogra et al., 2017) and a 5’UTR *acvr2bb* MO. Knockdown of Acvr2bb in MZ-*acvr2ba^−/−^* embryos lead to complete dorsalization (Figure 3G,H,J,L). To assess the specificity of MO knockdown, we injected mRNA for the maternally mutated gene, finding rescue of C5 dorsalized embryos to WT or a mild C1/C2 phenotype (Figure 3C,E,I,K). These results indicate that both maternal and zygotic expression of the Acvr2b receptors is key to DV embryonic axial patterning.

### Maternal and zygotic loss of Acvr2ba and Acvr2bb circumferentially expands dorsal cell fates at the expense of ventral and abrogates pSmad5

**Figure 4:**
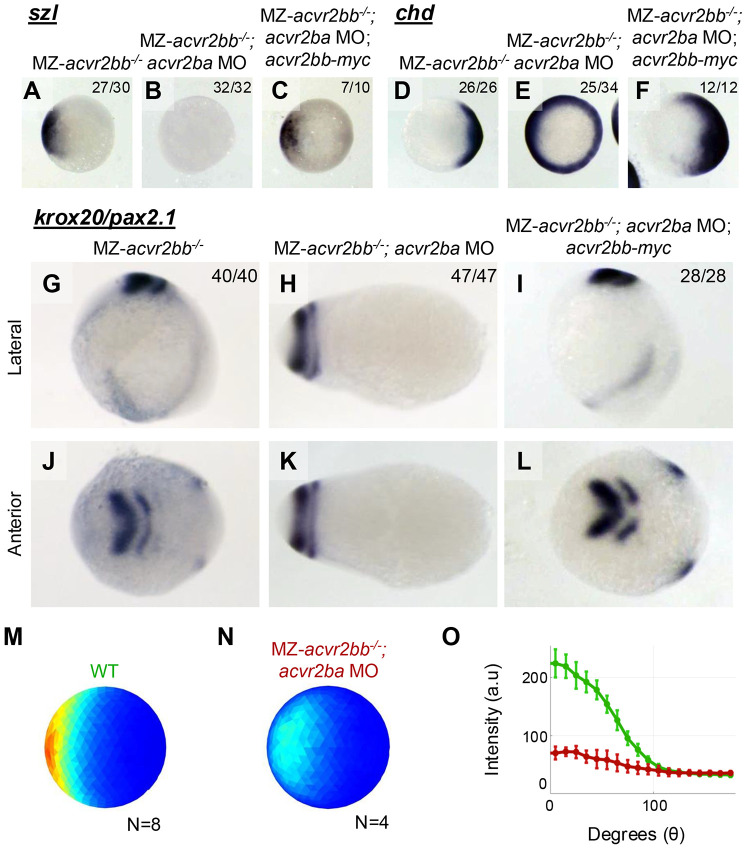
MZ depletion of Acvr2ba/bb results in molecular dorsalization of embryos Representative *in situ* hybridization for *szl* (A-C) *chd* (D-F) and 60% epiboly stages and *krox20/pax2.1* at 1-3 somite stages. *szl* marks the ventral mesoderm (A) and is absent in MZ*acvr2bb* embryos injected with *acvr2ba* MO (B). *chd* is expressed dorsally (D) and circumferentially expanded in MZ*acvr2bb* embryos injected with *acvr2ba* MO (E). 8/34 embryos displayed expanded, but not circumferential *chd*. Both domains are rescued by *acvr2bb* mRNA injection (C, F). Dorsal (G,I) and lateral (J,L) view of *pax2.1* marking the mid-hindbrain boundary (G,J anterior stripe) and *krox20* marking rhombomere 3 at 1-3 somite stage (G,J posterior stripe). In H and K, the embryo was rotated 90 degrees, but there is no clear lateral or anterior plane. Circumferential domains for these markers indicates expansion of the dorsal domain in MZ*acvr2bb* embryos injected with *acvr2ba* MO (H,K) that can be rescued with *acvr2bb* mRNA (I,L). Number of embryos displaying the observed phenotype of the total assayed, representative of 3 experiments (2 for C and F). Quantification of average phosphorylated-Smad5 levels in each nucleus from immunostained embryos at shield stage (M-O). Average pSmad5 gradient at the margin, error bars represent standard deviation.

To determine if maternal-zygotic loss of Acvr2ba and Acvr2bb abrogates BMP signaling in the gastrula, we examined the expression of DV cell fate markers using *in situ* hybridization. We focused on MZ-*acvr2bb* mutants with *acvr2ba* MO. Depletion of both Acvr2b receptors resulted in circumferential expansion of the dorsal marker *chordin* (*chrd*) (Miller-Bertoglio et al., 1997) and absence of the ventral BMP target gene *sizzled* (*szl*) (Yabe et al., 2003) (Figure 4A,B,D,E), consistent with a complete loss of BMP signaling. *acvr2bb* mRNA rescued *szl* expression and suppressed *chrd* expansion (Figure 4C,F), confirming that dorsalization is the result of Acvr2b loss. We additionally examined the expression of *pax2.1*, a midbrain-hindbrain boundary marker (Krauss et al., 1992), and *krox20*, a marker of rhombomere 3 at the 1-3 somite stage (Oxtoby and Jowett, 1993) (Figure 4G,J). MZ-*acvr2bb^−/−^* embryos with *acvr2ba* MO displayed a radial expansion of these markers, indicative of complete dorsalization of the embryo due to expanded dorsal neurectoderm, which was rescued by wild-type *acvr2bb* expression (Figure 4H,I,K,L).

To complement characterization of DV patterning changes, we investigated how MZ depletion of Acvr2b altered the gradient of phosphorylated Smad5 (pSmad5). We collected wild type and MZ-*acvr2bb^−/−^* embryos injected with *acvr2ba* MO at an early gastrulation (shield) stage, and performed quantitative analysis of pSmad5 nuclear levels in each cell of the embryo, as previously described (Zinski et al., 2017). Wild-type embryos displayed strong pSmad5 gradients (Figure 3M,O); however, MZ-*acvr2bb* mutant embryos with *acvr2ba* MO displayed a strong reduction in pSmad5 levels across the DV axis (Figure 4N,O). These results taken together demonstrate that maternal and zygotic contributions of Acvr2b receptors (Acvr2ba and Acvr2bb) mediate all the gastrula BMP signaling in the zebrafish.

### ACVR1-R206H requires maternal and zygotic contributions of both Acvr2ba and Acvr2bb to signal

**Figure 5:**
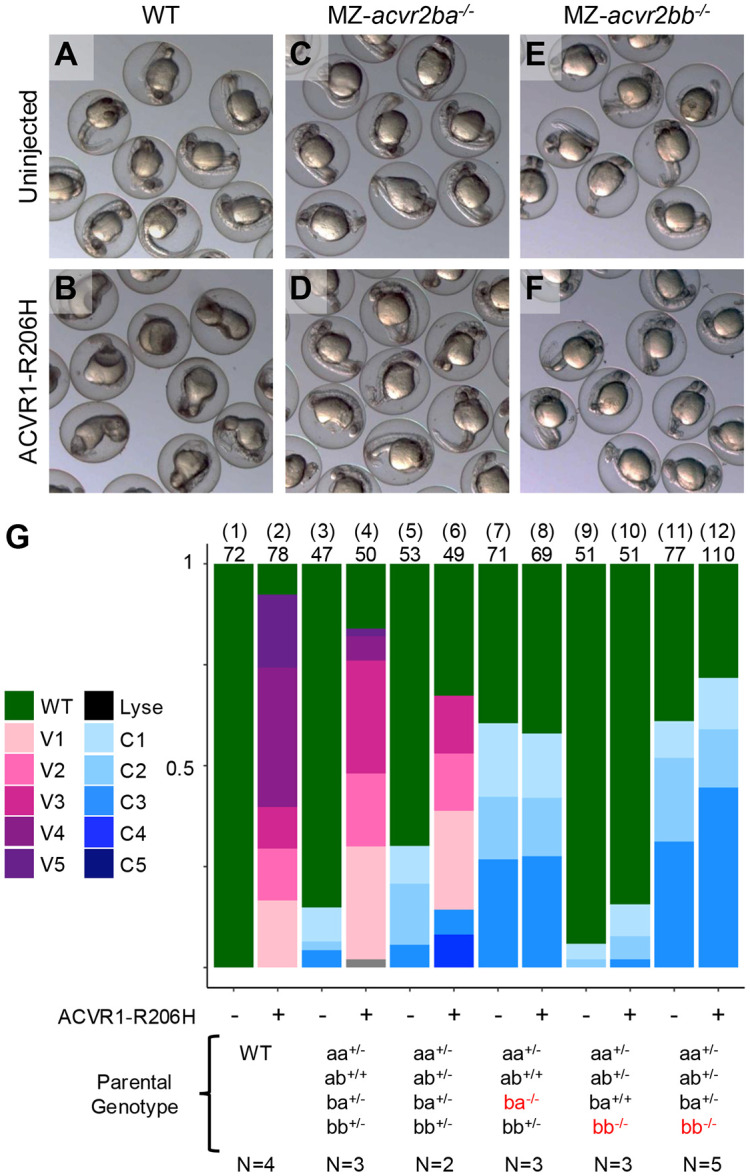
Dose dependent requirement for both Acvr2b receptors in FOP ACVR1 signaling Representative images of 1dpf embryos from wild type (WT) (A) and MZ-*acvr2ba* and MZ-*acvr2bb* embryos displaying mild dorsalization (B,C). Injection of ACVR1-R206H leads to severe ventralization of WT embryos (D) but does not change the phenotypes of MZ-*acvr2ba* or MZ-*acvr2bb* embryos, indicating a failure to induce hyperactive signaling without the maternal and zygotic pools of either receptor. Proportions of embryos displaying different phenotypes with or without ACVR1-R206H expression from different crosses (G), with maternal-zygotic mutant alleles highlighted in red. N indicates total number of individual crosses examined, number above bar indicates number of individual embryos examined, number in parentheses identifies column.

A recent study found that MO knockdown of both Acvr2ba and Acvr2bb in zebrafish reduced signaling by the FOP disease gene ACVR1-R206H that causes ectopic bone formation in humans (Lalonde et al., 2023); however, knockdown of Acvr2b did not completely abrogate signaling as expected if these receptors mediate all ACVR1-R206H signaling. Given our finding that maternal and zygotic Acvr2b receptors must be deficient to abrogate BMP signaling, we asked if these receptors were required for ectopic ACVR1-R206H signaling. We expressed ACVR1-R206H mRNA in either wild-type embryos (Figure 5A,B,G columns 1-2), MZ-*acvr2* mutants (Figure 5C-F,G columns 7-12), or exclusively in zygotic *acvr2* mutant embryos (Figure 5G columns 3-6). We found that ACVR1-R206H ventralized embryos from wild type and *acvr2* heterozygous crosses (Figure 5A,B,G columns 1-6), as expected. Interestingly, maternal and zygotic loss of either Acvr2ba or Acvr2bb prevented ectopic ventralization (Figure 5C-F,G columns 7-12), indicating that reduced Acvr2b receptor pools restrict ectopic signaling. In support of this, MZ-*acvr2bb^−/−^* embryos with completely wild-type maternal and zygotic contributions of Acvr2ba did not display ectopic signaling, indicating that loss of just one Acvr2b receptor limits ACVR1-R206H hyperactivity (Figure 5G, columns 9-10). As MZ-*acvr2bb^−/−^* mutants (Figure 5G column 9) are phenotypically wild-type, this suggests that the concentration of Acvr2ba present is sufficient for normal BMP signaling, but restrictive for ACVR1-R206H ectopic signaling. These results support a model in which Acvr2b receptors are limiting for FOP ACVR1-R206H hyperactivity in a dose-dependent manner.

## Discussion

While a majority of components of the BMP signaling mechanism acting in the zebrafish gastrula have been previously defined (Bauer et al., 2001, 2001; Kishimoto et al., 1997; Little and Mullins, 2009; Mintzer et al., 2001; Mullins et al., 1996; Nguyen et al., 1998; Schmid et al., 2000), the critical type II BMP receptors in this context were unknown. While recent evidence suggested that zygotic Acvr2 receptors facilitated signaling during dorsoventral patterning, only a minor reduction in BMP signaling was observed when these receptors were deficient zygotically (Preiß et al., 2022). As such, another source of type II receptors must contribute to signaling, either maternally deposited receptor pools or a redundant receptor able to signal in the absence of Acvr2, such as Bmpr2. We do not detect Bmpr2 transcripts in the early embryo (Figure 1), indicating that Bmpr2 does not play a role. Rather, we show that maternal Acvr2ba and Acvr2bb play major roles in transducing BMP signaling in DV axial patterning during gastrulation, together with zygotic expression of these two receptors. MZ-*acvr2ba*;*acvr2bb* mutant embryos display a loss of pSmad5 and circumferential expansion of dorsal fates at the expense of ventral cell fates in the embryo, reflecting a complete loss of BMP signaling. Our data show that Acvr2b receptors are the predominate type II receptors for BMP signaling in the gastrula and further indicate that endogenous levels of Acvr2a are insufficient for patterning. Thus, the BMP signaling mechanism in the zebrafish gastrula is comprised of a Bmp2/7 heterodimer that signals through a receptor tetramer consisting of Acvr1l, Bmpr1a, and a pair of Acvr2b receptors, with Acvr2a receptors playing a minor role in signaling.

Acvr2 receptors are type II receptors shared by both BMP and Nodal signaling, so may facilitate signaling through Smad5 and Smad2/3, respectively (Zinski et al., 2018). Surprisingly, zygotic mutation of all four Acvr2 genes results in a slight reduction in the BMP driven pSmad5 gradient, while Nodal induced pSmad2/3 is modestly increased in an unknown manner (Preiß et al., 2022). We find that maternal and zygotic loss of Acvr2b genes leads to complete embryonic dorsalization, in line with loss of all BMP signaling, but observe no phenotypes indicative of Nodal loss. In our experiments, the persistence in Nodal signaling may result from maternal Acvr2aa/ab contribution. However, the absence of nodal related phenotypes in MZ-*acvr2aa^−/−^*;*acvr2ab^−/−^* embryos suggests that Acvr2b receptors can facilitate Nodal signaling in addition to BMP signaling, suggesting that both Acvr2a and Acvr2b receptors facilitate Nodal signaling. Hence, maternal and zygotic depletion of all Acvr2 genes may be needed to eliminate early Nodal signaling in the embryo.

We previously demonstrated that Acvr1l kinase activity is necessary for signaling in the gastrula, though the kinase activity of the other required type I receptor, Bmpr1a, is dispensable (Tajer et al., 2021). Why only one receptor kinase acts in signaling when two receptors are available, and what facilitates the specialized kinase use through Acvr1l in this context, remain important questions in understanding BMP signaling. One possibility is that Acvr2b receptors are only capable of activating Acvr1l in the signaling complex. This could be regulated by receptor complex preformation, a process in which receptors form ligand-independent dimers (Agnew et al., 2021; Gilboa et al., 2000; Nohe et al., 2002). It has recently been proposed that preformed receptor dimers are essential precursors for active signaling complexes that allow type I receptors to adopt a poised state for activation (Agnew et al., 2021). Type I receptors compete for type II receptor binding in preformed complexes and preformation has been shown to determine which receptor signals. For instance, ACVR1 and ACVR1B (an Activin/Nodal-specific type I receptor) compete to form a complex with ACVR2A. Whether ACVR1 or ACVR1B complexes with ACVR2A determines whether SMAD1/5/9 or SMAD2/3 is activated, respectively (Szilágyi et al., 2022). Further studies will be needed to test if Acvr2b is uniquely positioned to phosphorylate Acvr1l, but not Bmpr1a, either through selective preformation or if the sub-functionalized kinase activity of Acvr1l is regulated via another mechanism.

Hyperactive signaling via the FOP disease-causing ACVR1-R206H further underscores the importance of Acvr2b receptors for signaling in the embryo. Maternal-zygotic loss of either Acvr2ba or Acvr2bb rendered ACVR1-R206H unable to signal, even when all three other Acvr2 receptors were present. This contrasts with Acvr2ba and Acvr2bb functioning redundantly in BMP signaling during DV patterning, where loss of both receptors is necessary to abrogate signaling. These findings are consistent with a dosage model in which reduced Acvr2b restricts ACVR1-R206H signaling but is sufficient for wild-type Acvr1l signaling. Further, a recent study found that ACVR2B, but not ACVR2A, preforms heterodimers with ACVR1-R206H that facilitate ligand-independent hyperactive signaling, consistent with our findings that Acvr2a is dispensable for ACVR1-R206H hyperactivity (Szilágyi et al., 2024).

In all, our findings reveal a key function for Acvr2ba and Acvr2bb in mediating all BMP signaling in the zebrafish gastrula. Further study of if and how Acvr2b regulates sub-functionalized type I receptor kinase activity, either by selective preformation with Acvr2b or through interactions with additional factors that delineate exclusive kinase activity through Acvr1l, is needed to understand how the receptor landscape drives interpretation of key signaling pathways for embryonic patterning.

## Methods

### Zebrafish husbandry

The research performed here was approved by the University of Pennsylvania Animal Care and Use Committee. Adult fish were housed at 28°C in a facility on a 13/11 light/dark cycle and cared for in accordance with institutional regulatory standards. Embryos were collected from crosses within 15 minutes and reared in E3 media (4 mM NaCl, 0.17 mM KCl, 0.33 mM CaCl2, and 0.33 mM MgSO4) at 28-31.5°C. DNA from adult fin clips or embryos was extracted via HotShot and used for genotyping (Meeker et al., 2007).

### Phenotypic evaluation

Embryonic phenotypes were assessed at 1 dpf based on previously established criteria (Kishimoto et al., 1997; Mullins et al., 1996; Nguyen et al., 1998). Embryos were imaged in E3 using a Leica IC80HD camera and images were processed in FIJI (Schindelin et al., 2012). In brief, C1 embryos partially lack the ventral tail fin, C2 do not have a ventral tail fin nor ventral tail vein, C3 embryos lack a yolk extension, C4 embryos have fewer than 13 somites, and C5 embryos exhibit strong dorsal convergence, evidenced by elongation at 12 hours post fertilization and lysis by 1 dpf. For ventral staging, V1 embryos are characterized by reduced head size, V2 lack notocords and have further reduction in head size, V3 lack anterior head structures structures and have slightly expanded somites, V4 display an exaggerated yolk extension and wider somites, and V5 embryos retain only somites. For genotyping, embryos were sorted by phenotype and then isolated into 96 well plates in methanol, preventing bias in scoring, before HotShot lysis to extract DNA.

### Microinjection of mRNA and MO

Embryos were collected within 15 minutes of fertilization in E3 media and kept at 22°C during injections. For each experimental replicate, a single needle containing either mRNA or MO was prepared and injected into all embryos on the same day. For conditions with multiple injections, all needles were prepared at the same time and serially injected into embryos, allowing independent assessment of the effect of each reagent on its own. Injection mixes were prepared such that 1-2nL yielded concentrations reported in Table 1, with a final concentration of 0.1M KCl and 0.1% phenol red. mRNA was synthesized from sequenced plasmids using the Sp6 mMessage mMachine kit from Ambion. Previously published *acvr2bb* MO (Dogra et al., 2017) led to off target head necrosis at concentrations that had no other phenotypes, so we designed a new 5’UTR targeting morpholino and injected a mix of both *acvr2bb* AUG MO and 5’UTR MO to increase specificity while reducing toxicity.

### CRISPR/Cas9 sgRNA generation

We mutagenized *acvr2aa*, *acvr2ba*, and *acvr2bb* using CRISPR-Cas9. Targets were selected with the assistance of the now discontinued CRISPR targeting resource on (http://crispr.mit.edu/) or the web tool CHOPCHOP ((https://chopchop.cbu.uib.no) (Montague et al., 2014). *acvr2aa* and *acvr2ba* Single Guide RNAs (sgRNAs) were synthesized in-vitro from PCR amplified templates using the cloning free method used in Gagnon et al 2014 (Gagnon et al., 2014), with the variation that sgRNA templates were amplified using Phusion Polymerase (ThermoFisher F553S), using the kit protocol scaled to a reaction size of 40μL. sgRNA templates were purified using the MinElute purification kit (QIAGEN 2800) to a final volume of 20μL. sgRNAs were synthesized from templates in-vitro using the MEGAshortscript T7 kit (ThermoFischer AM1354), using 8uLs of undiluted template in a reaction volume of 20uL. MEGAshortscript reactions were run overnight as this was found to increase yield. sgRNAs were purified using ethanol precipitation, as in Gagnon et al., 2014. Purified sgRNAs were assessed and quantified by running dilution series on a glyoxal/sodium phosphate buffer RNA gel and comparing to RiboRuler (ThermoFischer SM1821). *acvr2bb* sgRNAs were ordered directly from IDT rather than synthesized from DNA oligos. sgRNA sequences are presented in Table 2.

### CRISPR injection and allele identification

Immediately prior to injection, 2.5μL of undiluted sgRNA were mixed with 1μL of 5mg/mL Cas9-NLS protein (PNA Bio CP01-50), 1μL of Phenol Red, and 0.5μL 1M KCl (total volume 5μL) and kept at room temperature. 2nL of this injection mix were injected into wild type embryos. For *acvr2bb*, guides were injected into triple *acvr2aa*;*acvr2ab*;*acvr2ba* heterozygous incrosses. To identify mutant alleles, F1 DNA samples were cleaned with ExoSapit (AppliedBiosystems) and sequences were extracted from heterozygous trace data with the help of the web app PolyPeakParser (Hill et al., 2014).

### Genotyping

Kompetitive allele specific PCR amplification (KASPar) genotyping was used to genotype *acvr2aa*, *acvr2ab*, and *acvr2bb* (Table 3). A PCR band size difference was used to genotype *acvr2ba* mutants (162 bp wild-type band and 241 bp mutant band) using the standard NEB 2xTaq protocol modified to include 3% DMSO (12.5 μL 2xTaq, 1 μL 10 μM forward and reverse primer, 0.75 μL DMSO, 5.25 μL water, 5 μL gDNA). Primers used for genotyping *acvr2aa*, *acvr2ba*, and *acvr2bb* are reported in Table 3.

For *acvr2ab*, the sequence provided to LGC genomics was:

TGGAGAAAAGGACAAGCGRCGACACTGTTTCTCCACCTGGAAGAACCGCT[C/A]GGGRACCATCGAGATGGTSAAGCAGGGCTGCTGGCYGGACGATGTCAACT.

### in situ *hybridization*

Whole mount *in situ* hybridization was performed on shield, 60%, 1-3 somite, and 17 somite stage embryos using DIG labeled anti-sense probes (made with the Roche 11277073910 kit), following the multi-basket protocol established in (Thisse and Thisse, 2008), though using no proteinase K for any stage. Probes for *pax2.1* (Krauss et al., 199*2)*, *krox20* (Oxtoby and Jowett, 1993), *szl* (Yabe et al., 2003), and *chd* (Miller-Bertoglio et al., 1997) were generated previously, while *bmpr2b* was made from a full length CDS plasmid. Probes for *bmpr2a* were synthesized from genomic DNA PCR products using a primer set containing a T7 sequence: F – CTCAGAGTCCTGCAATGCCA, R – taatacgactcactatagggGTCAAGTCCTCGTTGGCTGA.

### Immunofluorescent staining and quantification

pSmad5 immunostaining and quantification was performed as described (Zinski et al., 2019). Briefly, shield stage embryos were fixed in 4% formaldehyde in PBS with 0.1 % TritonX (PBST) O/N at 4°C, washed and deyolked manually, then incubated in a 10% FBS, 1% DMSO in PBST blocking solution. Samples were then incubated O/N at 4°C with a 1:200 dilution of rabbit pSmad1/5/9 antibody (Cell Signaling 13820) in block, washed 3x with PBST, then incubated O/N at 4°C in 1:500 dilution of anti-rabbit Alexa 647 (Invitrogen-21245) and 1:2000 sytox green (Fisher S7020) in block, followed by 3-4 20 minutes PBST washes. Immunostained embryos were then dehydrated in methanol and cleared using a 1:2 ratio of benzyl alcohol (Sigma B-104) and benzyl benzoate (Sigma B-6630) to match the refractive index of yolk. Embryos were mounted with the DV axis parallel to the cover slip and imaged using a Ziess LSM880 confocal with LD LCI Plan-Achromat 25x/0.8 lmm Corr DIC M27 multi-immersion objective set to oil-immersion. 2 WT embryos were included for each experiment to account for differences in staining and laser intensity across sessions and averaged to account for changes across the imaging session.

Image files were converted to TIFFs and nuclei were segmented by Sytox staining. The pSmad5 intensity for each nucleus was calculated. The mean intensity for each WT embryos was calculated, and for each experiment the WT embryos were averaged to generate a scaling value so that the average WT intensity was the same. This scaling value was applied to all embryos imaged on the same day to normalize staining and imaging differences. Resulting embryos were aligned across the DV axis and aligned to a reference, WT embryo. Population means were generated across each experimental group and nuclei were projected into equilateral triangles tiling the embryo to generate heatmaps. Margin gradients were calculated by examining a 30μm band of cells at the margin in 10 degree increments (Zinski et al., 2019).

### Analysis of published datasets

RNA-seq data from (White et al., 2017) was downloaded from https://www.ebi.ac.uk/gxa/experiments/E-ERAD-475/Results by searching for Gene ID and exporting transcripts per million (TPM) at each developmental stage. The heatmap in Figure 4was generated from TPM data using the R package, pheatmap (Raivo Kolde, 2010).

### Generation of plots

Stacked boxplots were generated using ggplot2 (Wickham, 2016). pSmad5 intensity and margin gradient plots were generated using a published MatLab analysis pipeline (Zinski et al., 2019).

## Supplementary Material

Supplement 1

## Figures and Tables

**Table 1:** Injection constructs and concentrations

	Sequence	Concentration
*acvr2ba* MO	TGAGCAGAGAAGCGAACATATTCCT	2.5-5ng
*acvr2bb* MO (ATG)	AGCCAGCCAGGGAACAAACATATTC	2.5ng
*acvr2bb* MO (5’UTR)	GTGCGTCTTTATGCCGTGAAAGCAT	5ng
*acvr2ba-myc*	Full length CDS	50pg
*acvr2bb-myc*	Full length CDS	10-50pg
*ACVR1-R206H*	As in (Allen et al., 2020)	120-240pg

**Table 2:** CRISPR guides

	sgRNA
*acvr2aa*	GGGCACGATGGAGTACAGAAGGG
*acvr2ba*	GGAGCCGGAGTTGTTCCTCCAGG
*acvr2bb*	GAGCGGGTTCGAGAGGTGCGAGG

**Table 3:** Genotyping primers

Gene	Forward primer 1	Forward Primer 2	Reverse Primer
*acvr2aa*	GAAGGTGACCAAGT TCATGCTCATGATG GGCACGAt	GAAGGTCGGAGTCA ACGGATTCATGATG GGCACGAa	CCCAGAAGCCGCCA CTGTTCAGTAC
*acvr2ba*	GAAAGACAAACGCT CGCACTGC	N/A	GTTGTCGAGCCGGT GCTCATATAATTC
*acvr2bb*	GAAGGTGACCAAGT TCATGCTGCGGGTT CGAGAGGt	GAAGGTCGGAGTCA ACGGATTGCGGGTT CGAGAGGg	CGCGTAACAGTGAA GCCTCTTATCT

