# Supplementary material for: Acvr2b receptors transduce all BMP signaling in the zebrafish gastrula and restrict Fibrodysplasia Ossificans Progressiva ACVR1-R206H signaling in a dose-dependent manner
Source: bioRxiv. 2025 Oct 7:2025.10.07.680911. Preprint. [Version 1] doi: 10.1101/2025.10.07.680911 (PMC12632381; doi:10.1101/2025.10.07.680911)
Supplement: Supplement 1 [file media-1.pdf]

Figure S1: Acvr2 mutants result in frameshifts with premature stop codons

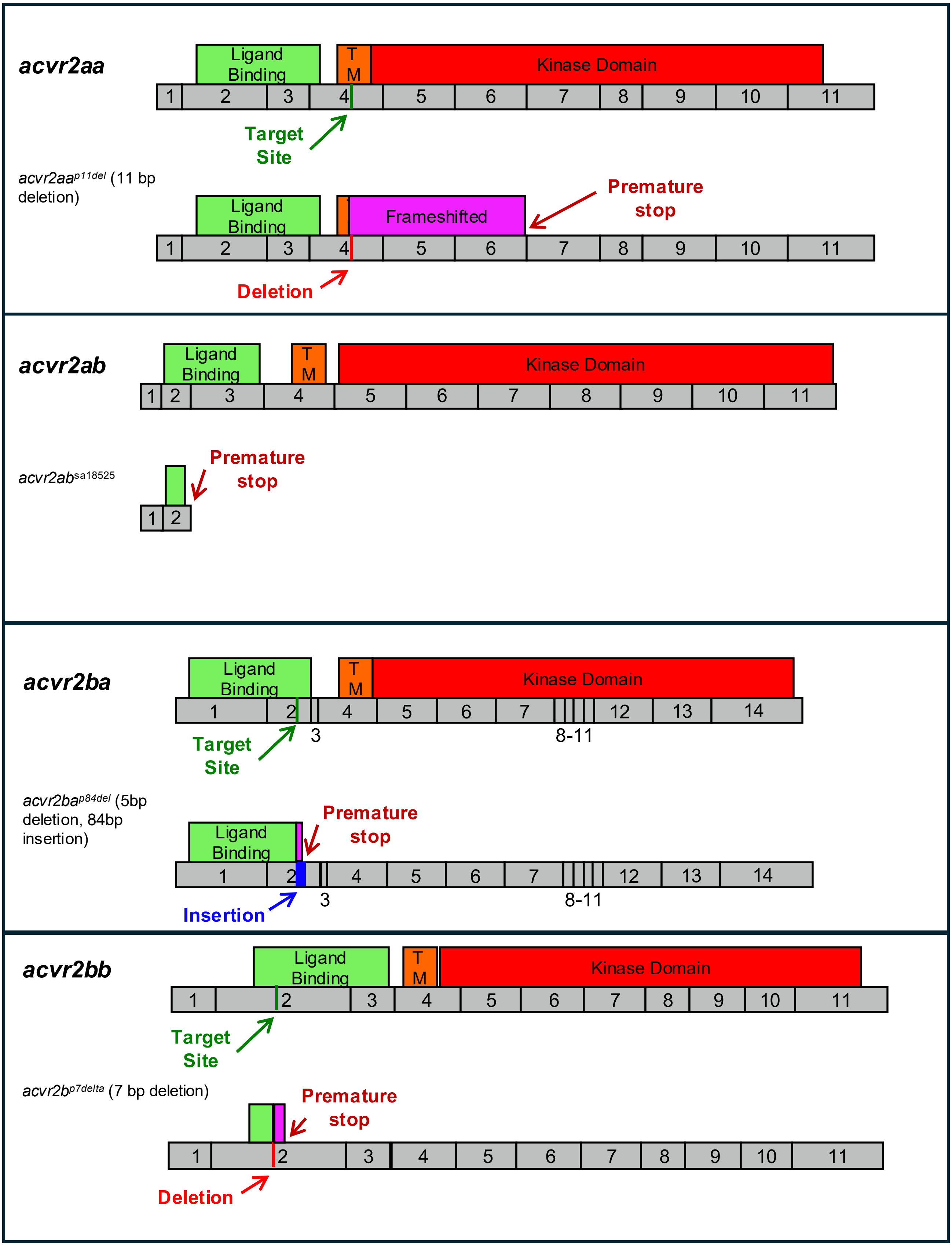

Figure S1: Acvr2 mutants result in frameshifts with premature stop codons
List of *acvr2* alleles generated with CRISPR or obtained from ZIRC, and their positions relative to the CRISPR target site (green), exon structure (grey), and major protein domains: Ligand binding domain (light green), transmembrane domain (orange), and kinase domain (red). Insertions shown in blue. Presumptive modifications to the transcript or protein are represented in purple.
